# Getting the pieces to fit: NHS and third sector collaboration to enhance crisis mental health service provision for young people

**DOI:** 10.1186/s12913-023-09198-w

**Published:** 2023-03-30

**Authors:** Bobbie Dutton, Neil Humphrey, Pamela Qualter

**Affiliations:** The University of Manchester, Manchester Institute of Education, University of Manchester, Oxford Road, M13 9PL Manchester, England

**Keywords:** Collaboration, Working together, Third sector, VCSE, Mental health, Crisis services

## Abstract

**Background:**

The increase in demand for young people’s mental health services has been met by a growth of co-located mental health service provision in the NHS and third sector. This research explores the benefits and challenges of the NHS collaborating with a charity to provide a step-down crisis mental health service for young people in Greater Manchester, and suggests how the collaboration between the NHS and third sector may be improved for future projects.

**Methods:**

Working from a critical realist paradigm, this qualitative case study utilised thematic analysis of 9 in-depth interviews with operational stakeholders from 3 operational layers, to explore insiders’ perspectives of the benefits and challenges of collaboration between the NHS and third sector in the context of the ‘Safe Zones’ initiative.

**Results:**

Themes relating to perceived benefits of collaboration were: doing things differently, flexibility, a hybrid approach, shared expertise, and shared learning. These were counterbalanced by perceived challenges: getting the pieces to fit, obtaining a shared vision, geography, lack of referrals, and timing. The importance of effective communication (e.g. of shared vision, standard operating procedures, key performance indicators) was noted as central to addressing challenges and reaping benefits.

**Conclusions:**

NHS and third sector collaboration can yield a range of benefits, some of which can mitigate against the perceived inflexibility and restrictive nature of usual mental health service provision, thereby providing a vehicle for innovation in step-down crisis care for young people.

**Supplementary Information:**

The online version contains supplementary material available at 10.1186/s12913-023-09198-w.

## Background

Only 25% of children and young people (CYP) who experience clinically significant mental health difficulties actually receive specialist treatment [1]. It is unsurprising, therefore, that there has been a sharp rise in mental health related accident and emergency (A&E) attendances by this age group in recent years [2], as increasing numbers of them experience a crisis, defined by the National Health Service (NHS) as,“occurring when the level of distress and risk presented by a young person is not adequately supported or contained by the system that is in place for them.” [3].

The increasingly high prevalence rates of mental health difficulties among CYP have applied mounting pressure upon A&E and in-patient facilities across the UK [4, 5]. However, there is an increasing amount of evidence which suggests that A&E departments are not appropriate or effective in handling mental health crisis [6], and that most CYP would rather not attend an A&E when in crisis [7]. Acknowledging this, recent policy (e.g. Crisis Care Concordat [8] & Five Year Forward View for Mental Health [9]) reflects a recognition that crisis mental health services need serious investment and renovation. As a result of devolution, Greater Manchester has been able to control its health and social care spending to address the current short falls of mental health crisis services for CYP, with the creation of the Crisis Care Pathway (CCP).

### Policy context

Despite the key policy initiatives set out by the UK government (e.g. No health without mental health [10], the Health and Social Care Act 2012 [11], the Crisis Care Concordat [8], Future in Mind [1]), the Five Year Forward View for Mental Health highlighted that the increased demand for mental health services and system wide challenges for implementing ‘parity of esteem’ had led to inadequate provision and deteriorating outcomes of mental health services, including an increase in suicide rates [9]. It was within the context of these policies that the CCP was created to provide a service pathway that was accessible to all CYP across Greater Manchester who experience a mental health crisis.

### The crisis care pathway

The CCP was created by Greater Manchester Health and Social Care Partnership (GMHSCP) to alleviate the growing pressures upon emergency services and to improve the experiences of CYP undergoing crisis. The CCP is multifaceted and continues to evolve as it is rolled out throughout the 10 boroughs of Greater Manchester. At the time of this research, the pathway included seven new or enhanced services: Liaison Mental Health, the Rapid Response Team, Assessment Centre, Enhanced In-Patient Provision, Medical on Call, Enhanced Community CAMHS Cover, and Safe Zones. The aim was that these services work together, and with the wider health and social care system, in order to address the current systems failures and provide a comprehensive service pathway for CYP when experiencing a mental health crisis.

The new pathway reflects recent policy (see Policy Context) by (1) attempting to link services together more closely and work together in an integrated way; (2) providing urgent access to mental health services for CYP in crisis; (3) ensuring 24/7 access to crisis services; (4) having a fully supported stepped down approach from crisis; (5) improving the experience of CYP and their families accessing crisis services; (6) treating CYP in the community where possible; (7) reducing pressures on emergency departments; (8) providing a needs led approach; and, (9) and offering a range of services to meet a diverse range of needs [3].

### Safe zones

Whilst most of the services within the CCP are new, or a newly enhanced version of an old service, they can be traced back to similar services from Adult Mental Health Services (AMHS). Examples include the Rapid Response Teams, which parallels with Crisis Response Home Treatment Teams, and Liaison Mental Health, which already exists in hospitals for adults. Safe Zones, on the other hand, represents a novel service model which offers a blended approach between youth work and traditional clinical models. The service has been designed for young people aged 13 to 18 years who are experiencing a mental health crisis, or have recently had a crisis de-escalated, but are not in need of any immediate medical attention [12]. At the time of this research, young people were only able to access the service through referral from the Rapid Response Team (another service in the CCP). However, the long-term goal is that CYP will be able to access Safe Zones through other routes (e.g. drop in). Safe Zones have four working sites across Greater Manchester, which are classified as the North, Central, South and West sites respectively. Once at Safe Zones, service users can receive one to four sessions of needs-led, tailored support, and leave with a clear care-plan, knowing where to find further help if necessary [13].

The service operates in lines with the CCP’s aims of preventing unnecessary hospital attendance and paediatric ward admittance for CYP in MH crisis, but also signifies a new approach of working together, by collaborating with the third sector to provide this novel service. Safe Zones is the first service within Greater Manchester’s CAMHS to be sub-contracted to the voluntary, community and social enterprise (VCSE) sector. It has been sub-contracted by Pennine Care NHS Foundation Trust, on behalf of GMHSCP, to The Children’s Society, who work in partnership with other local charities, Bolton Lads and Girls Club, Manchester Youth Zone, and 42^nd^ Street, to deliver their services [14]. Although there is a partnership between these charities to provide the services (e.g. utilise the partners workforce and spaces), The Children’s Society is the lead partner and coordinator of Safe Zones.

### Research aims

The aim of this research is to understand the benefits and challenges of collaboration between the NHS and third sector to provide crisis care for young people, and to make recommendations for future collaboration efforts.

## Method

### Paradigm and positionality

This research has been developed within a critical realist paradigm. It is understood that this research explores a set *real* phenomenon (e.g., the NHS sub-contracting Safe Zones to the third sector to deliver crisis mental health services), but with the appreciation that our understanding of this phenomenon is shaped through context specific subjective experiences of it. Although, “human knowledge captures only a small part of a deeper and vaster reality” (p. 182), there is no other way of exploring the world [15]. Therefore, in-depth semi-structured interviews with Safe Zones’ operational stakeholders were considered the most appropriate method to answer the research questions set out in this study.

The research team holds a range of experience, with the second and third author having over 30 years of combined research experience in mental health. The first author, who conducted the interviews and led the analysis process, has personal experience of accessing crisis services, and later worked with young people in educational settings with mental health difficulties, before starting her PhD researching crisis services. This study is part of a PhD research, which evaluates the CCP that Safe Zones is a part of. As such, the lead researcher worked closely with project leads at GMHSCP, visiting service sites, attending board meetings, and keeping in regular contact with funders and providers.

### Sample

Participants were recruited from across three operational layers of Safe Zones, in order to gather a range of perspectives about the benefits and challenges of the collaboration efforts. In total, nine operational stakeholders participated in this study: five staff working within Safe Zones who deliver services to young people; two leaders within The Children’s Society who made key contributions to Safe Zones development and/or delivery; and two CCP leads from the NHS who were involved in the oversight of the initiative. Although participation in this research was kept anonymous, participants would know of one another and work together in different capacities, depending on what operational layer they worked within. Participant’s professional experiences ranged, but not reported in this research to uphold anonymity.

Safe Zones only has a small dedicated workforce, ergo, a small population to pull from. Nine participants was deemed as a satisfactory sample size for this research, as it pulled from all three operational layers. Participants ranged in terms of demographics. Personal data regarding age, gender identification, sexuality, and ethnicity was not collected to preserve anonymity, and bore no relevance to the research’s objectives.

As a relationship was already established with senior staff within the CCP, the selection of participants was determined through purposeful sampling, making use of professional contacts already established for the CCP leads from the NHS. Then, utilising the Safe Zones project manager as a gate keeper to contact the remaining participants, who did not have a prior relationship with the evaluation team. Participants were then sent relevant research information sheets and opt-in consent forms, which provided the full details of the research. Once consent forms were received, interviews were then arranged. Out of all the participants who consented to take part in the research, no one dropped out.

### Interviews

Semi-structured interviews were used to generate data and were conducted by the lead researcher. The lead researcher has received training in, and experience of conducting interviews as part of her MSc in Research Methods and PhD, alongside her previous role as a research assistant. Due to the COVID-19 lockdown restrictions, interviews were held via Zoom in personal residences with no one else in attendance beside the interviewer and the participant. Interviews were audio recorded for later transcription. On average, interviews lasted 50 min. No repeat interviews were carried out.

The interview schedule and questions were created by the research team (BD, NH, and PQ), with help from members of The Children’s Society, to ensure that questions were deemed fair and appropriate for the research. Interview schedules were standardised. Participants were given the opportunity to expand on issues which they felt more important to them, and less on questions which they felt were less relevant to their experience. Once interviews were concluded, participants were given time to ask any follow up questions and thanked for their time.

### Thematic analysis

The six phases of thematic analysis, outlined by Braun and Clarke [16], were used in this research. The lead author conducted the interviews and transcribed them, which assisted with the first step of *familiarizing yourself with the data.* This helped provide a *feel* for what the participants were saying, before the analysis process had even begun. Being privy to the development of Safe Zones (by attending monthly board meetings, visiting Safe Zones sites, and regularly communicating with service providers) also helped with the familiarisation process, as the lead author could quickly understand what participants were communicating from prior knowledge of the context.

An inductive approach to analysis was taken, starting with the development of semantic codes for the second phase: *generating initial codes *[16]. This process was led by the first author, which was then reviewed by the second and third author, to ensure the names given for the themes unambiguously represented the original data. Themes were then generated from these initial codes.

Once themes had been generated, the reviewing process begun. As Braun and Clarke continue to reiterate, the six phases of thematic analysis outlined in their 2006 paper are not a rigid framework [16]. This flexibility was particularly useful at this stage, as from the fourth phase (*reviewing themes*), there was movement both forward to phase five (*defining and naming themes*), and backwards to phase three (*generating themes*). Being able to move back and forth enabled reflection about whether the themes generated were relevant to the aims of this research.

### Trustworthiness

This research adheres to Lincoln and Guba’s trustworthiness criteria [17] for qualitative research. Credibility was established through prolonged and persistent engagement with Safe Zones and the wider CCP, and a *thick description* of the research context has been described so that findings may be transferred to other similar settings [18]. The research design and methods were developed with input from the CCP and The Children’s Society to ensure that all parties were satisfied with the final design. Interviews were also *member-checked* by participants, and initial findings were shared with available participants and leads from Safe Zones and the CCP, to sense check and provide a space for them to provide evaluative feedback of the analysis process to ensure dependability with findings. NVivo was utilised for the thematic analysis of the interviews, keeping an easily accessible audit trail amongst the research team.

### Ethical approval and consent to participate.

This study was reviewed and signed off by the first author’s supervisory team at The University of Manchester, and GMHSCP, the commissioners of the CCP evaluation. Standard ethical principles were followed: gaining informed consent from participants; providing participants the option to withdraw from the study; anonymization of data; and, following General Data Protection Regulation guidelines. As all participants were professionals who were interviewed about their profession, it was unlikely that their involvement in these interviews would cause distress or harm.

## Results

### Organisation

Themes developed from analysis of the interview data are presented pragmatically to clearly focus on the aims of this research; understanding the benefits and challenges of collaboration from the perspectives of operational stakeholders. For this reason, themes relate directly to challenges and benefits and are organised under these umbrella categories. See Table 1 for a summary of themes for the perceived benefits of collaboration, and see Table 2 for a summary of themes for the perceived challenges of collaboration.

**Table 1:** Collaboration benefits

Themes
Doing things differently
Flexibility
A hybrid approach
Shared expertise
Shared learning

**Table 2:** Collaboration challenges

Themes
Getting the pieces to fit
Obtaining a shared vision
Geography
Lack of referrals
Timing
The importance of communication

More benefits and challenges were reported by participants than have been relayed in this paper. Those that appeared few times were either categorised into larger themes or coded within a “miscellaneous” category, which is not reported here. The themes developed within this paper interlink closely with one another; therefore, they have been written to display the narrative of the overall picture of collaboration.

In their interviews, participants would often interchange the language utilised regarding The Children’s Society. They would swap between referring to working with “The Children’s Society”, “charities”, “VCSE’s”, the “third sector”, and more. As the “third sector” embodies all of these, this term is adopted for this paper.

### The perceived benefits of collaboration

#### Doing things differently

Sub-contracting Safe Zones was unanimously recognised by participants as a positive form of collaboration between the NHS and the third sector. All participants reported that by doing this, they were able to *do things differently* compared to traditional mental health services typically provided within the NHS for young people.

“If we had of put that [Safe Zones] into the NHS we wouldn’t have had the same opportunities of what we could offer the young people” (Interview 6)

Participants felt that sub-contracting Safe Zones outside of the NHS to a third party, in this case The Children’s Society, created the space to *do things differently* in comparison to the status quo of CYP crisis services within the NHS, meaning that they could be more innovative when providing crisis care for young people (see theme *hybrid approach*).

#### Flexibility

The ability to* do things differently* within Safe Zones was attributed to *flexibility,* which came from the sub-contracting relationship. It was perceived by participants that by sub-contracting Safe Zones to The Children’s Society, who operate within the third sector, the service was not constrained by the same ‘red tape’ as those services which operate within the NHS.

“Just the absolute mind-bending nature of interfacing between the NHS, which is a behemoth, it’s got its own way of doing things […] with the third sector, which are much more used to being flexible; going with the flow, picking up small projects and then dropping them again, and then being very needs-led.” (Interview 8)

This *flexibility* created an opportunity to *do things differently* with crisis services, leading to Safe Zones providing an innovative service, which would not have been feasible within the confines of traditional crisis services in the NHS.

#### A hybrid approach

Safe Zones was considered to be an innovative service as it was able to draw upon traditional therapeutic roots held within the NHS, but also the youth work expertise of the third sector, creating a *hybrid approach*.

“[Safe Zones] offer something slightly different than just a clinical model. So having a blended approach of both youth work and clinical approaches, so young people have the time and space to explore what they can do for themselves for self-help and their mental health. So rather than it just being around medication and things like that, it’s actually about getting them involved in other things. So that could be in sports and that could be creative, it could be gardening, anything really.” (Interview 1)

Therefore, Safe Zones could provide a service that was less intense than traditional clinical therapy, but which was more intense than common youth work provision, leading to a service which was able to provide “needs-led” care for young people in crisis.

“We offer kind of a really interesting mix of structured interventions around goals, but at the same time the way that they’re structured are different for every single young person.” (Interview 8)

It is clear from participant’s responses that being able to draw on traditional therapies and youth work through this *hybrid approach* meant that they could easily tailor their care to the individual needs of young people accessing their service.

#### Shared expertise

The *hybrid approach* was made possible through the *shared expertise* of the NHS and third sector. Safe Zones was able to provide, “DBT and CBT informed therapies… around self-esteem, confidence [and] anxiety”, whilst also tapping into The Children’s Society’s “expertise and knowledge” in youth work to create bespoke “needs-led” sessions with young people. However, it is not just the systems which bring *expertise*, it is the people who work within these systems. Unlike traditional MH crisis services within the NHS, Safe Zones was able to draw staff from a wider range of professional backgrounds. This included qualified practitioners who would likely be found in other NHS mental health services, but also experts from the third sector, such as youth workers and people with experience in drama and art therapy.

#### Shared learning

Participants, particularly those who worked on the ground within Safe Zones, reported the benefits of this “mixing pot” of *shared expertise*, leading to a *shared learning* for those working within the service.

“I think that’s quite nice being able to have that space to not just hire trained therapists. It has meant that there’s just a whole different wealth of experience and everyone does come at things really differently and I feel like I’ve learned so, so much from different people.” (Interview 4)

Not only has this led to *shared learning*, but also created a “very supportive environment” for staff working within Safe Zones. Participants saw this as a key benefit, as it meant they were able to receive support from peers to better support their work with young people entering the service.

“The amount of support that I’ve had from the seniors that I work with has been brilliant […] it’s a massive help because you share knowledge and ideas of how to progress your work.” (Interview 4)

S*hared learning* was particularly pertinent during the COVID-19 pandemic. During this time, face to face services were disrupted, and alternative methods of service delivery were needed. As The Children’s Society had experience of setting up telephone mental health services previously, they were able to share this process with the CCP and adapt services swiftly to provide remote support.

“[Safe Zones] being able to draw on experience really is a positive. If you take the example of the well-being phone line that’s been set up to support COVID, that’s been a real benefit because they’ve just built onto an existing service that they’ve had, so in that area their experiences are really valuable and it’s a real benefit.” (Interview 9)

The entire CCP were able to learn from this *shared expertise* to create telephone lines which spanned the rest of the service pathway. All services within the CCP were affected during pandemic, either due to limitations with face-to-face contact with service users, or the need for staff to work from home. Therefore, not only did *shared learning* benefit Safe Zones, it also went on to benefit other CYP MH crisis services within the NHS.

### The perceived challenges of collaboration

#### Getting the pieces to fit

The clearest collaboration challenge which was evident throughout participant’s interviews was the bewildering complexity involved in mixing the two worlds of the NHS and the third sector and *getting the pieces to fit*. Participants described them as two separate entities with their own working styles and different processes, which appeared to be difficult to combine at times.

“The big one is not to assume that our policies and procedures actually fit in correctly with NHS provision. As a charity you’ve got reams and reams of processes in place, but actually, when you start to look at what the NHS work with, you realise you’re not quite there.” (Interview 1)

Although the third sector have strict safeguarding processes in place, the NHS required more. The *flexibility* of the third sector, whilst seen by participants as a significant benefit of the collaboration, was at odds with the “red tape” of the NHS and its strict procedures. Compromise was identified as being a key feature in the ability of the NHS’s and third sector’s effort to *get the pieces to fit* properly.

“Trying to interface the NHS way of thinking about things and the third sector way of thinking about things […] does not easily fit. The puzzle pieces are completely different shapes, so I think the NHS have had to round off some of their sharp edges and we’ve had to sharpen up some of ours so we can actually interface with each other.” (Interview 8)

However, it has been through this challenge that *shared learning* was able to take place, to create the *hybrid approach,* which is led by young people’s need, becoming a key benefit in the collaboration process.

“It’s also a benefit, in that the NHS are using a model and practitioners that they would never use before with youth work and needs led […] it’s also a really amazing opportunity for the charity as well, to really firm up their understanding of what is required of a mental health service and clinical governance and quality assurance. So it kind of cuts both ways.” (Interview 8)

Nonetheless, the challenge of *getting the pieces to fit* led to other significant challenges in the implementation of the Safe Zones service, which could be attributed to a challenge identified by most participants; *obtaining a shared vision*.

#### Obtaining a shared vision

*Obtaining a shared vision* appeared to be a challenge when establishing Safe Zones. Participants working within the service shared their ‘on the ground’ experiences, highlighting the realities of how *getting the pieces to fit* can appear in practice.

“I think it can sometimes be a bit disjointed, if I am being perfectly honest. I think because we have so many different people involved […] I think it can get quite messy.” (Interview 8)

Participants connected this messiness with what was referred to as struggling to attain a “shared vision”, “shared understanding”, or “getting everyone on the same page”. Therefore, *obtaining a shared vision* appeared to be a key challenge, which underpinned two other key challenged highlighted by participants; *lack of referrals* and *geography*.

#### Geography

As Safe Zones is a part of the CCP, which was designed to serve all of Greater Manchester, a great deal of consideration was made regarding their positioning across the 10 boroughs of GM. In Safe Zones’ Standard Operating Procedure (SOP), a minimum of four sites was considered necessary to run across GM effectively. However, during the implementation of Safe Zones, the opening of the fourth site was delayed significantly due to problems securing a fit location for the service. This meant a substantial part of Greater Manchester’s *geography* was not being covered, right up until the beginning of the UK lockdown, when all sites needed to be closed and switch to distance working and alternative service provision. Whilst this proved to be a challenge by its own right, the tension surrounding the geography of Safe Zones’ sites ran deeper, illustrating the complexities of *obtaining a shared vision* amongst all parties.

The *geography* of the Safe Zones sites was clear within the SOP that it should be a minimum of 4, which is what was planned for within the budget given by The Children’s Society and ultimately produced. Although on the surface this would appear that a *shared vision* had been obtained regarding the *geography* and spread of the Safe Zones sites, interviews uncovered tensions between participants understanding of this. Some believing that:

“Given in the big scheme of things a fairly limited budget […] we had good geographic coverage over Greater Manchester.” (Interview 2)

But most saw the *geographical* coverage as a place where Safe Zones could improve or grow in the future. One of the more interesting discoveries was that being from the same level of operations did not ensure a *shared vision*. Only two participants could be interviewed from the third operational level, both of whom were CCP leads within Safe Zones operation, and yet still they differed in their perceptions towards the *geography* of Safe Zones. One shared the opinion of most, that Safe Zones have provided what they could within the limited budget and time and that expansion was something that could come in the future.

“I think in terms of the place that works for you that feels a bit of an unfair question to some extent because we’re talking about an enormous geography. […] One of the things that maybe in future phases is […] more sites to enable a higher spread of zones across the geography.” (Interview 9)

However, the other participant from the same operational layer questioned the process by which the partnership of Safe Zones was actioned by The Children’s Society, believing that this impacted the *geography* and spread of Safe Zones. This participant expressed the belief that they have not lived up to the original *vision*.

“I think the vision initially was that this partnership would go in and create a way to get priority access into some of those services […] not only just use the physical space but actually give us a menu of options across Greater Manchester for services we could put those young people forward.” (Interview 6)

Instead, Safe Zones made use of their partners’ spaces and created their own team of trained professionals. Whilst this participant believed this team was “brilliant” and this way of working brought many “pros”, it did not live up to the original *vision* set forth by the funders.

#### Lack of referrals

Another challenge that was underpinned by the difficultly of *obtaining a shared vision* was the *lack of referrals* into Safe Zones during the implementation of the service. Referral routes were an obvious source of tension between the commissioning body and sub-contractors of Safe Zones. There was a phased implementation of Safe Zones, so after waiting several months for the organisation and finalisation of contracts, they had a further six months of limited referral routes. The *lack of referrals* not only made it difficult to prove the value of Safe Zones, but also affected the morale of the work force. It was frustrating for those working within the service as they felt that they were able to work with more young people than were being referred into them.

“I would say it got very frustrating when… the referrals hadn’t picked up, so I was waiting for a while for my young people to work with, so that was very frustrating and then I kind of felt that I was a bit of a fraud because […] I’m waiting and I’m waiting and I’m waiting, and I just felt really like, you know when you get a bit like you run out of batteries, you just run out of all enthusiasm?” (Interview 7)

Whilst CCP leads (those responsible for the oversight of Safe Zones) recognised that the *lack of referrals* was through no fault of the service, they also understood this would affect their ability to prove their impact. The limit on referrals was justified in order to protect Safe Zones from becoming oversubscribed and unable to meet demand; overall protecting the greater good of the CCP.

“They’ve not used their capacity within the hours they are available and that’s frustrating for them, […] it’s a bit of a precarious tight rope between their capacity suddenly flipping on its head and them not being able to meet the demand […] it feels harsh to say that’s a weakness because it’s absolutely not their fault, but I think it’s probably something that will let them down in terms of being able to review the impact of the service, which is a shame.”(Interview 6)

The *lack of referrals* caused tension between the funders and sub-contractors, creating a challenge for Safe Zones to prove their impact and cost effectiveness. Once again, this highlights the challenge of *obtaining a shared vision*, but this time regarding the outcomes expected from the service, and how they will be measured.

#### Timing

The final significant challenge identified by participants was *timing*. The challenges discussed all contribute to the first, *getting the pieces to fit*, and this takes *time* (more time than expected). The NHS and The Children’s Society took much more time than expected working out the complexities of their contract, leading to a domino effect of other aspects of implementation taking more time.

“Because our contract was delayed […] then our sub-contracts were then delayed with our partners, […] managing all that detail really that takes up time, quite rightly, but you know, we’ve got to get it right.” (Interview 2)

Whilst the delays were considered the product of a necessary process to create solid foundations for the service, the delayed start eventually contributed to the concern that Safe Zones were *running out of time* to prove their worth as a service. As Safe Zones was a pilot, those working within knew they had a *limited amount of time* to prove the success, which the delayed start compounded. Participants expressed a belief that the main way to prove their success was by having more young people access their service, and they would need *more time* to do this:

“The immediate word that comes to mind for that is “more time”. To really get the best understanding and to enable it to succeed it needs more time to get more people through the door to allow more review and then to allow more future thinking and innovation.” (Interview 9)

Participants believed the way in which to prove their success was related to the amount of young people they could serve, but the *lack of time* due to delays, and the *lack of referrals* made this task all the more challenging.

#### The importance of communication

When considering how to overcome one of the key challenge raised above*, obtaining a shared vision*, it is clear that ensuring all parties are “on the same page” is a necessity. But collaboration involves two or more parties working together, and in the case of Safe Zones, two different systems where, *getting the pieces to fit*, can be challenge. Therefore, it is no surprise that participants reported that *communication* across the service has also been a challenge.

“For example [Person K] might have an idea that isn’t communicated to the rest of us, or not in time, and something is agreed that we didn’t know about, and then it trickles down in a really odd way, [Person E] doesn’t find out about it, and I think communication can be quite tricky.” (Interview 8)

Although from an outsider’s perspective, the solution of better *communication* routes may seem obvious, from participants’ interviews it is clear that they believed this was going to be an inherent challenge of the collaboration from the start.

“I mean, you guys have got 4 different trusts involved at that kind of level, it’s bonkers. And all these people have to stay on the same page as each other, that’s always going to hinder quick progress.” (Interview 8)

Clear systems of *communication* were already in place within Safe Zones through regular meetings and correspondence at all operational levels, but this is clearly an area for improvement, and a lesson for future collaborators; a clear system of *communication* does not guarantee a *shared vision*. Therefore, messages and visions being *communicated* need to be tempered to realistic expectations of their potential interpretations, and that key foundations to these expectations are clearly recorded in relevant, transparent documentation.

## Discussion

The aim of this research was to understand the benefits and challenges of collaboration between the NHS and third sector to provide step-down crisis MH services to CYP in Greater Manchester, and what could be learnt from this experience for future collaborative endeavours. We used thematic analysis to analyse 9 semi-structured interviews with stakeholders from across 3 operational layers of the Safe Zones service. Analysis of participant interviews established 5 themes which could be considered benefits of collaboration: *doing things differently, flexibility, a hybrid approach, shared expertise,* and *shared learning.* The 5 themes categorised as challenges to collaboration were; *getting the pieces to fit, obtaining a shared vision, geography, lack of referrals,* and *timing*. Finally, the importance of *communication* was discussed as a final theme, highlighting the importance of understanding in the communication process, and having realistic expectations of shared visions when collaborating in complex systems.

Collaboration has been a strong theme pushed throughout recent NHS policy development, including more recently in the NHS Long Term Plan that promotes “a new way of working” through bringing people, resources, and capabilities together to deliver “greater value for the NHS and for patients” [19]. There has been a growing global demand for Integrated Care [20], and was a key vision set out in the Five Year Forward View, which aspired to bring traditionally divided NHS services together [21]. Integrated Care has continued to be at the forefront of service provision and innovation, and has strengthened relationships between the NHS and other partners in local communities, including the voluntary, community and social enterprise (VCSE) sector (referred to in this study as the third sector).

Collaboration is promoted through NHS policies, specifically in guidance on Integrated Care, by encouraging partnerships between the NHS and local organisations and agencies within communities [22, 23]. However, how this looks in practice can be extremely variable. This variability is most probably an outcome of a well-intended, yet problematic, feature of NHS policy surrounding collaboration, which is that *what* these collaborative partnerships should look like, or *how* to establish them effectively, it is not well defined. This is to allow local health providers to establish partnerships which are targeted to address the needs of their communities [19]. Our findings support the key message within policy documents that support and encourage collaboration [22]: it is fruitful endeavour that reaps many benefits. Nevertheless, whilst third sector and NHS partnerships are strongly encouraged, little evidence exists about the results of collaboration in practice, particularly from operational stakeholders’ perspectives. The current case study begins to fill the knowledge gap regarding how the NHS and third sector collaborate in practice, to create an entirely new and novel MH crisis service within the NHS.

A key benefit identified within this research has been the ability of Safe Zones to *do things differently*, which has come from the *flexibility* of the third sector. Whilst efforts have been made centrally and locally within the NHS to ease the burden of bureaucracy [24], there were clear undertones from participants that the NHS was viewed as retaining rigid structures and ways of working which prevented it from being able to provide innovative MH services for CYP. Alternatively, the third sector was viewed as a space where new and creative services could be implemented, without being restricted by the “red tape” of the NHS. In Safe Zones, this has led to the provision of *a hybrid approach* of crisis support which *shares expertise* of traditional therapies and youth work, creating an environment where *shared learning* can take place. Whilst this clearly demonstrates the positives of collaboration, it also points towards a deeper issue within the NHS, through a perceived lack of ability to be innovative or flexible with service development and provision.

Therefore, collaboration with the third sector could act as a mechanism to encourage creative innovation of services funded by the NHS. Alternatively, however, the NHS could take the opportunity to reflect upon their own systems of practice, which have been criticised by the participants in this research for being too bureaucratic and limited by red tape, and learn from the third sector to develop systems that are more flexible to allow for creative innovation. As the largest provider of MH services in the UK, the NHS is the first point of contact for crisis support for all ages, so it would make sense for them to be at the forefront of development of these services. As it appears that current systems are limiting them from achieving this, it may be time for a revaluation.

The final theme discussed in this paper, *the importance of communication*, echoes the NHS document ‘Understanding the key success factors in collaborative working’ [25] that establishes *harmonisation across cultures* as a major issue in collaborative working. Whilst *harmonisation across cultures* in the NHS document relates to the theme *getting the pieces to fit* in this research, the NHS document refers to challenges of recruitment, training and uniform policy and standards, whereas the findings of this research addresses a deeper conflict of working cultures and practices between the NHS and third sector. *Communication* is necessary for building strong and effective working relationships [26], and is a key contributor towards *getting the pieces to fit* together. However, *communicating* to *obtain a shared vision*, as demonstrated in this research, is not so easy to achieve in practice. Collaborators may be under the impression that they share the same vision, and have communicated regularly and effectively to achieve this, but the outcomes still vary. Therefore, key documents, such as the Standard Operating Procedure, and Key Performance Indicators, should be updated regularly to reflect the vision of the service, explain how this will be shared across all stakeholder, and how it will be evaluated. This will enable a fundamental vision to be shared, which is accessible to all involved in the provision of the service, although expectations of this vision need to be realistic.

### Limitations, strengths and future work

The implementation of Safe Zones, and the design of this research, was interrupted by the onset of the COVID-19 pandemic, and following lockdown. This caused major disruptions to the service, and required a change in the research design to become more flexible to the changes in COVID related restrictions and regulations. Participant’s responses may have also been effected by the impact of COVID on their personal and professional lives. This was a cross-sectional study, taking place during a difficult time of delivery. The authors of this paper believe that a longitudinal study would be helpful in understanding the benefits and challenges of collaboration over time and outside of the restrictions and effects of the pandemic. This study only included the perspectives of operational stakeholders and could be further enhanced by including the perspectives of service users, to gauge the impact of collaboration on YP accessing crisis services.

A real strength of the current research is the cross validation of the different layers of operation, providing a perspective from different localities and from each layer of the collaborative team. This increased the trustworthiness of the findings within this research, as it provides a multidimensional perspective of the case study phenomenon. Because the focus of this research was on the sub-contracted collaborative partnership developed within Safe Zones, the findings from our research may be transferred to other collaborative efforts across the NHS, not limiting it to young people’s crisis mental health services. However, because our focus was on the collaborative partnership, there was only scope to include professionals in this study. It is crucial to consider the perspective of service users in future work.

## Conclusion

This study has established that collaboration between the NHS and third sector to provide crisis MH care for CYP is not only feasible, but desirable, for the many benefits which it produces. Whilst this paper highlights the number of challenges faced during the process of collaboration in this case study, it also provides an opportunity for reflection on these challenges, and benefits, which we may learn from. A clear practical learning point which can be taken away from the analysis of the challenges, is the importance of understanding and expectations in communication. Describing a vision does not provide sufficient detail to perfectly replicate it, therefore expectations should be tempered and key foundations of the vision of the service should be made as clear as possible through recorded key documents, which are accessible to everyone and updated regularly.

On the other hand, analysis of the benefits of this collaboration unearth deeper reflections about the need for collaboration in the first place. The benefits are doubtlessly supportive of collaboration, but point towards a fundamental problem within the NHS’s provision of MH services. The participants of this study hail the third sector for its innovation and ability to do things differently, which ultimately emphasises the NHS’s perceived inability to do this. Two key implications of this are as follows: (1) The NHS should further enhance their efforts to collaborate with the third sector to develop and deliver their crisis MH services for CYP to prosper from the benefits of collaboration. (2) The NHS should reflect upon and reform current systems of practice which are limiting their ability to develop and provide innovative crisis MH services.

## Supplementary information

**Additional file 1.** Safe Zones: Interview Questions

## Data Availability

The datasets used and analysed during the current study are not available to ensure participants remain unidentifiable. Full transcript data may lead to participants becoming identifiable due to the size of the service under investigation.

## Abbreviations

A&E: Accident and Emergency
CAMHS: Child and Adolescent Mental Health Services
CCP: Crisis Care Pathway
CYP: Children and Young People
GMHSCP: Greater Manchester Health and Social Care Partnership
MH: Mental Health
NHS: National Health Service
SOP: Standard Operating Procedure
UK: United Kingdom
VCSE: Voluntary, Community and Social Enterprise
