# Supplementary material for: Getting the pieces to fit: NHS and third sector collaboration to enhance crisis mental health service provision for young people
Source: BMC Health Serv Res. 2023 Mar 30;23:307. doi: 10.1186/s12913-023-09198-w (PMC10061406; doi:10.1186/s12913-023-09198-w)
Supplement: Supplementary file 1 — Additional file 1. Safe Zones: Interview Questions [file 12913_2023_9198_MOESM1_ESM.docx]

**Safe Zones: Interview Questions**

1. What is the purpose of Safe Zones?
2. Why were Safe Zones created?
3. Are there any similar services like Safe Zones that you know of?
4. Do you think there is anything unique about Safe Zones?
5. What services do Safe Zones offer?
6. How would describe Safe Zones relationship with other services?
7. How does Safe Zones fit within the Crisis Care Pathway?
8. What is your understanding of a mental health crisis?
9. How would you describe your role within Safe Zones?
10. How would you describe the training for your role?
11. Do you feel supported in your role?
12. What other roles exist within Safe Zones?
13. Do you know what other roles do in Safe Zones?
14. How would describe your relationship with other roles within Safe Zones?
15. How would you describe the leadership within Safe Zones?
16. What feedback mechanisms exist within Safe Zones?
17. Do you think there has been a clear plan of implementation for Safe Zones?
18. Do you know who developed the plan of implementation for Safe Zones?
19. Who do you believe is responsible for implementing this plan?
20. How flexible has the plan been?
21. Can you talk me through the strengths and weaknesses of Safe Zones?
22. Do you think the model of Safe Zones works for delivering the outcomes set?
23. What do you believe the strengths and weaknesses of Safe Zones has been so far?
24. What external or internal factors may have helped or hindered the progress of Safe Zones?
25. What do you think would enable Safe Zones to improve their services?
26. What do you think the benefits and challenges of sub-contracting Safe Zones to The Children’s Society has been?
27. Is there anything else you would like to add?
